# Comparative Metabolomic Analysis of Different Organs of Understory-Transplanted and Wild *Dendropanax dentiger*

**DOI:** 10.3390/metabo16060354

**Published:** 2026-05-25

**Authors:** Jianshuang Shen, Yiyun Chen, Hang Zhang, Tianze Hu

**Affiliations:** 1 Hangzhou Polytechnic University, Hangzhou 310018, China; 2495101037@hzvtc.edu.cn (H.Z.); 2495101009@hzvtc.edu.cn (T.H.); 2 Wuchan Zhongda Changle Forest Farm, Hangzhou 310018, China; chenyy19@wzgroup.cn

**Keywords:** *Dendropanax dentiger*, understory-transplanted, untargeted metabolomics, metabolic components, organ specificity

## Abstract

**Background**: The artificial cultivation of *Dendropanax dentiger* under forest understory conditions offers a sustainable alternative to wild harvesting, yet the metabolic adaptations underlying transplantation stress and recovery remain poorly understood. Objectives: In this study, we performed a comparative metabolomics analysis of different organs (leaves, current-year stems, three-year-old stems, and roots) from wild *D. dentiger* plants and those transplanted to the understory. **Methods** and **Results**: Metabolite annotation and classification revealed that over 60% of the metabolites fell into the categories of lipids and lipid-like molecules, organoheterocyclic compounds, phenylpropanoids, and polyketides. Further differential analysis of metabolites showed that understory transplantation significantly altered the metabolic profiles of all organs, exhibiting organ-specific response patterns. For the metabolite components in the organs of transplanted and wild *D. dentiger*, these metabolites were mainly classified into eight categories: alkaloids and derivatives; benzenoids; lignans, neolignans and related compounds; lipids and lipid-like molecules; organic acids and derivatives; organoheterocyclic compounds; phenylpropanoids and polyketides; and organic oxygen compounds. Notably, the contents of (-)-asarinin, (Z)-1-(methylthio)-5-phenyl-1-penten-3-yne, and stearidonic acid (SDA, 18:4n-3) were higher in transplanted plants than in wild plants, indicating the potential of understory cultivation for the targeted extraction of these bioactive compounds. **Conclusion**: These findings provide a metabolomics basis for optimizing the artificial cultivation and quality control of *D. dentiger*. This study highlights the value of metabolomics in understanding the metabolic composition of *D. dentiger* and offers a reference for its artificial cultivation.

## 1. Introduction

*Dendropanax dentiger* (Harms) Merr., a small evergreen tree belonging to the genus *Dendropanax* within the family Araliaceae, is native to China and commonly found in broad-leaved mixed forests in valleys or on mountain slopes at altitudes ranging from 200 to 1400 m, with a natural distribution also extending to Southeast Asia. This species integrates medicinal, edible, ornamental, and nectar-producing values, making it a plant with high potential for development as both a food and medicinal resource. Its roots, stems, and leaves have been traditionally used in folk medicine for centuries to treat rheumatoid arthritis, migraine, and dysmenorrhea [1]. Modern research has confirmed that *D. dentiger* is rich in various active components, including flavonoids, saponins, and phenolic acids, which exhibit anti-inflammatory, antioxidant, and immunomodulatory pharmacological activities [2]. Regarding its edible value, the tender leaves are abundant in proteins, free amino acids, vitamin C, total flavonoids, and saponins while containing low levels of nitrate and nitrite, representing a high-quality woody vegetable resource [3]. Additionally, with its diverse leaf morphology and evergreen nature, *D. dentiger* can serve as an understory landscaping species and nectar source, offering significant ecological and economic value [4]. As market demand for medicinal and edible homologous products continues to grow, *D. dentiger* demonstrates broad application prospects in functional foods, traditional Chinese medicine, and landscaping.

Existing studies have confirmed the core value of *D. dentiger*, particularly its roots and leaves, as a medicinal and edible resource. The tender leaves are nutritionally rich, containing functional phytochemicals such as flavonoids and polyphenols, while exhibiting low nitrate and nitrite contents, making them a typical woody vegetable with dual medicinal and edible functions or therapeutic health benefits, warranting further development and utilization [3]. The roots of *D. dentiger* contain structurally diverse and bioactive compounds, including steroids, alkaloids, flavonoids, and monoterpenoids, some of which display anti-inflammatory, cytotoxic, and antioxidant activities [2,5,6,7], indicating potential applications in biomedicine, functional health foods, food additives, beverage formulations, and feed additives.

Although the resource value of *D. dentiger* has been widely recognized, there remains a lack of systematic comparative studies on whether the accumulation characteristics of medicinal components under cultivated conditions differ from those in wild conditions and whether the composition and content of metabolites are stable across different organs (roots, stems, and leaves). In recent years, untargeted metabolomics has been widely applied in the quality control and evaluation of natural products, crop quality improvement, resource identification, and the discovery of novel biomarkers [7,8,9]. This technique enables comprehensive and systematic qualitative and quantitative analysis of secondary metabolites in natural products, providing an effective approach to address the aforementioned issues.

Therefore, this study conducted a comparative untargeted metabolomic analysis of various organs (roots, stems, and leaves) of *Dendropanax dentiger* grown under understory transplantation versus wild conditions, systematically elucidating the differences in metabolite composition and accumulation characteristics. The aim is to reveal the mechanisms by which understory cultivation influences the formation of medicinal components in *D. dentiger*. The findings are expected to provide a scientific basis for transitioning *D. dentiger* from wild harvesting to understory ecological cultivation, thereby promoting the integration of forestry and health industries, supporting rural revitalization and sustainable ecological development, and converting the substantial development potential of *D. dentiger* into tangible economic and ecological benefits.

## 2. Materials and Methods

### 2.1. Plant Materials

The plant materials used in this study were *Dendropanax dentiger* collected from two locations in China. Wild *D. dentiger* samples were obtained from the Qianjiangyuan–Baishanzu Qingyuan Conservation Center of China National Park (119°14′01.23″ E, 27°46′16.25″ N; altitude 1107.2 m). Understory-transplanted *D. dentiger* samples were collected from a *Cunninghamia lanceolata* plantation at Jinzifeng (118°58′39.20″ E, 27°40′02.00″ N; altitude 1105.2 m). On 7 October 2025, leaves (the first and second leaves from the apex), current-year stems, three-year-old stems, and roots were separately collected from each location. All samples were immediately frozen in liquid nitrogen and stored at −80 °C for subsequent analysis.

### 2.2. Untargeted Metabolite Profiling and Data Analysis

#### 2.2.1. Sample Preparation and Sequencing

Samples were sent to Novogene Biological Information Technology (Beijing, China) for untargeted metabolome sequencing via liquid chromatography–mass spectrometry (LC–MS), with six biological replicates per sample type. The sample codes were designated as follows: for understory-transplanted plants, leaf (leaf1), one-year-old stem (stem1_1year), three-year-old stem (stem1_3year), and root (root1); for wild plants, leaf (leaf2), one-year-old stem (stem2_1year), three-year-old stem (stem2_3year), and root (root2). Tissue specimens (100 mg each) were separately pulverized with liquid nitrogen, and the resulting homogenate was suspended in 500 μL pre-cooled 80% methanol containing 0.1% formic acid by vortexing, following the method described by Shen et al. [10].

UHPLC-MS/MS analyses were performed using a Vanquish UHPLC system (ThermoFisher, Germering, Germany) coupled with an Orbitrap Q Exactive^TM^ HF mass spectrometer, Orbitrap Q Exactive^TM^ HF-X mass spectrometer, Orbitrap Exploris™ 120 mass spectrometer, or Orbitrap Exploris™ 480 mass spectrometer (ThermoFisher, Germany) at Novogene Co., Ltd. (Beijing, China). Samples were injected onto an ACQUITY UPLC BEH Amide Column (Milford, MA, USA) (100 × 2.1 mm, 1.7 μm) using a 12 min linear gradient at a flow rate of 0.2 mL/min. The eluents for the positive and negative polarity modes were eluent A (5 mM ammonium acetate in 90% ACN) and eluent B (5 mM ammonium acetate in 50% ACN). The solvent gradient was set as follows: 2% B, 1.5 min; 2–100% B, 7 min; 100% B, 9 min; 100–2% B, 9.1 min; 2% B, 12 min. The Q Exactive^TM^ HF mass spectrometer was operated in positive/negative polarity mode with a spray voltage of 3.5 kV, capillary temperature of 320 °C, sheath gas flow rate of 35 arb, aux gas flow rate of 10 arb, S-lens RF level of 60, and Aux gas heater temperature of 350 °C.

The data files generated by UHPLC-MS/MS were processed by XCMS v4.9.0 to perform peak alignment, peak picking, and quantitation for each metabolite [11]. Then, based on adduct ions and the mass deviation set to 10 ppm, a comparison was made between these data and the self-built high-quality secondary spectrum database (NovoMetDB) to obtain results for metabolite identification. After eliminating background ions according to blank samples, the original quantitative results were normalized by the following formula to obtain relative peak areas: Relative peak areas = Raw quantitative value of samples/(The sum of quantitative value of samples/The sum of quantitative value of QC1). Compounds with a coefficient of variation (CV) of relative peak areas in QC samples greater than 30% were removed. Finally, the identification and relative quantitation results of metabolites were obtained. Data processing is based on the Linux operating system (CentOS version 6.6), using R v4.5.2 and Python.v 3.14.0 For details on specific packages and software versions used, readers are referred to the README file included in the Results.

#### 2.2.2. Data Preprocessing and Metabolite Identification

Raw MS data were converted to mzXML format using ProteoWizard v3.0 [12] and subsequently processed with XCMS software [13] for peak extraction, alignment, and retention time correction. Total peak areas within each sample were normalized, and peaks with missing rates exceeding 50% across all groups were filtered out. Metabolite identification was performed by searching the filtered results against the Novogene in-house database.

Data preprocessing was performed using XCMS software. Metabolite identification was achieved by matching the acquired data against a high-quality MS/MS spectral database based on parameters such as mass accuracy and adduct ion information. Metabolites with a coefficient of variance (CV) less than 30% in QC samples [14] were retained for further analysis. Peak areas of each feature were integrated using XCMS, and the relative quantification of each metabolite was normalized by total peak area.

#### 2.2.3. Multivariate Statistical Analysis

For multivariate statistical analysis, principal component analysis (PCA) and partial least squares discriminant analysis (PLS-DA) were conducted to reveal differences in metabolic profiles among groups. Hierarchical clustering analysis (HCA) and metabolite correlation analysis were performed to explore relationships among samples and metabolites. Functional analysis, including metabolic pathway enrichment, was carried out to interpret the biological significance of the identified metabolites.

The Pearson correlation coefficient between QC samples was calculated based on the relative quantification values [15]. PCA was performed using all peaks extracted from both experimental and QC samples. Functional annotation and classification of the identified metabolites were carried out using the KEGG, HMDB, and LIPID MAPS databases. Data from positive and negative ion modes were combined for subsequent analyses.

#### 2.2.4. Screening of Differential Metabolites

Differential metabolites were screened based on three parameters: variable importance in projection (VIP) from the PLS-DA model [16], fold change (FC), and *p*-value. VIP values indicate the contribution of each metabolite to group separation. FC was calculated as the ratio of the mean relative quantification values of each metabolite between comparison groups. *p*-values were calculated using Student’s *t*-test [17] to assess statistical significance. Metabolites with VIP > 1.0, |FC| > 1.5, and *p* < 0.05 [18] were considered differentially accumulated metabolites.

#### 2.2.5. Visualization and Functional Analysis

Hierarchical clustering analysis (HCA) [19] was performed on all differential metabolites across comparison groups, with normalized relative quantification values used for clustering. Chord diagrams were generated based on correlation coefficients among differential metabolites to illustrate correlations and associations, with the top 20 differential metabolites (ranked by *p*-value) selected for visualization.

## 3. Results

### 3.1. Data Quality Control

**Figure 1:**
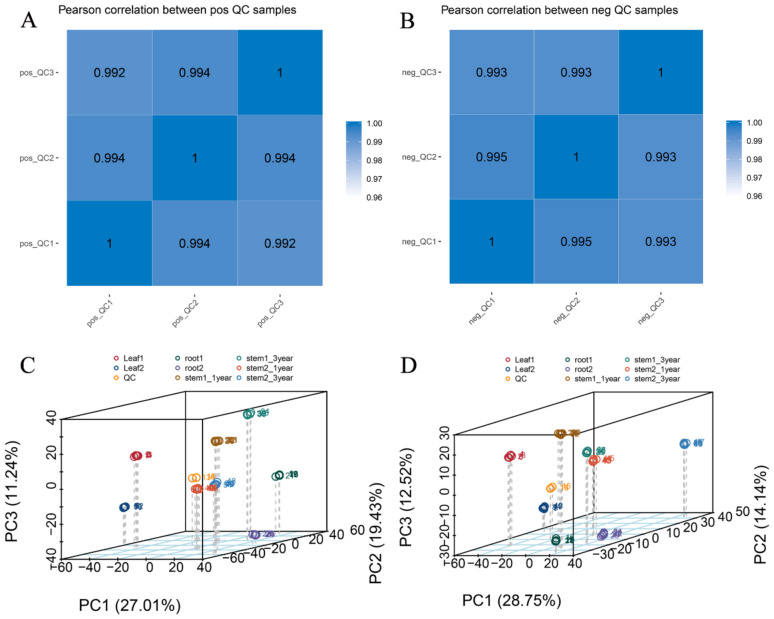
Pearson correlation analysis of QC samples and PCA plots based on the metabolic profiles of all samples: (**A**,**B**) Pearson correlation analysis in positive and negative ion modes, respectively. Each cell represents the correlation coefficient (R^2^) between two QC injections. (**C**,**D**) PCA plots based on the metabolic profiles of all samples in positive and negative ion modes, respectively. The explained variances for PC1, PC2, and PC3 are provided in parentheses on each axis.

Pearson correlation analysis (R^2^ > 0.992) performed on the quality control samples (Figure 1A,B) indicates that the method exhibits good robustness, the data quality is reliable, and the results are consistent with the PCA data (Figure 1C,D).

### 3.2. Metabolite Pathway and Classification Annotation

Metabolite analysis was performed on the leaves, one-year-old branches, three-year-old branches, and roots of wild and cultivated *D. dentiger*. The results showed that 6185 compounds (3423 in positive ion mode and 2762 in negative ion mode) were annotated using three metabolite databases (Table S1). The representative LC–MS chromatograms for each sample group are shown in Figures S1 and S2. Functional and classification annotation of the identified metabolites was carried out using major databases, namely HMDB (https://hmdb.ca/metabolites, accessed on 20 October 2025), KEGG (https://www.genome.jp/kegg/pathway.html, accessed on 20 October 2025), and LIPID MAPS.

**Figure 2:**
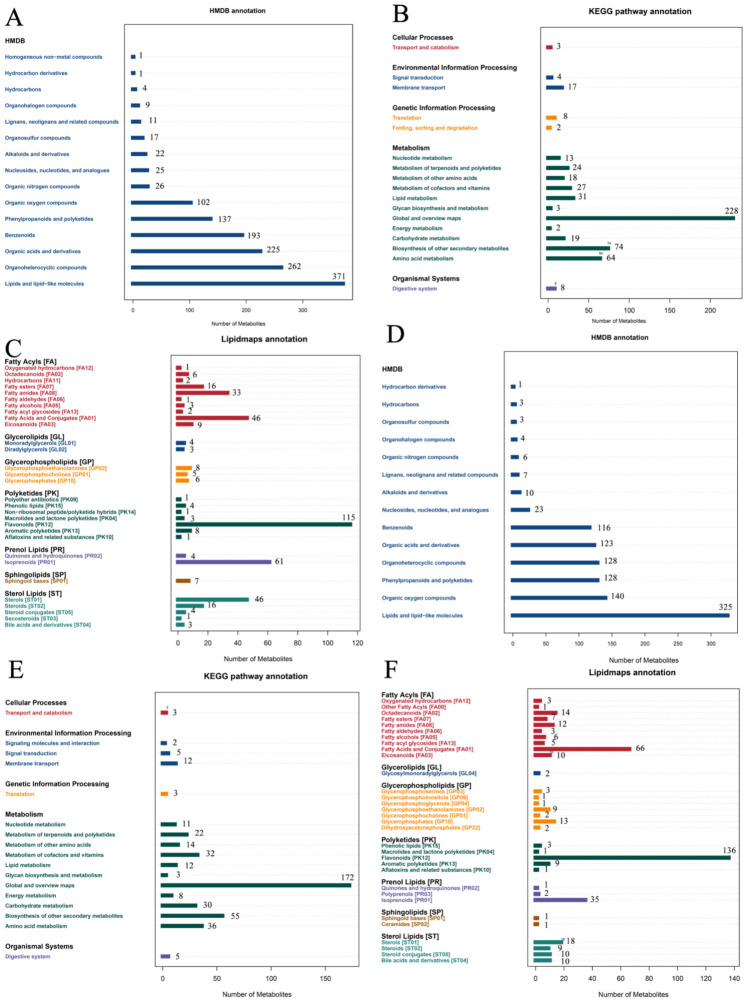
Metabolite annotation results based on HMDB, KEGG, and LIPID MAPS databases in positive and negative ion modes: (**A**–**C**) Metabolite annotation results in positive ion mode. (**D**–**F**) Metabolite annotation results in negative ion mode. (**A**,**D**) HMDB superclass annotation. (**B**,**E**) KEGG pathway annotation. (**C**,**F**) LIPID MAPS classification annotation. The horizontal axis represents the number of metabolites, and the vertical axis represents the annotated HMDB superclasses, KEGG pathways, or LIPID MAPS main classes.

In Figure 2, the annotation results from each database are summarized. Among them, the most frequently annotated categories were as follows: 696 compounds (371 in positive ion mode and 325 in negative ion mode) were assigned to the HMDB superclass “lipids and lipid-like molecules”; 400 compounds (228 in positive ion mode and 172 in negative ion mode) were annotated to the KEGG pathway “global and overview maps”; and 251 compounds (115 in positive ion mode and 136 in negative ion mode) were classified under the LIPID MAPS category “flavonoids”.

**Figure 3:**
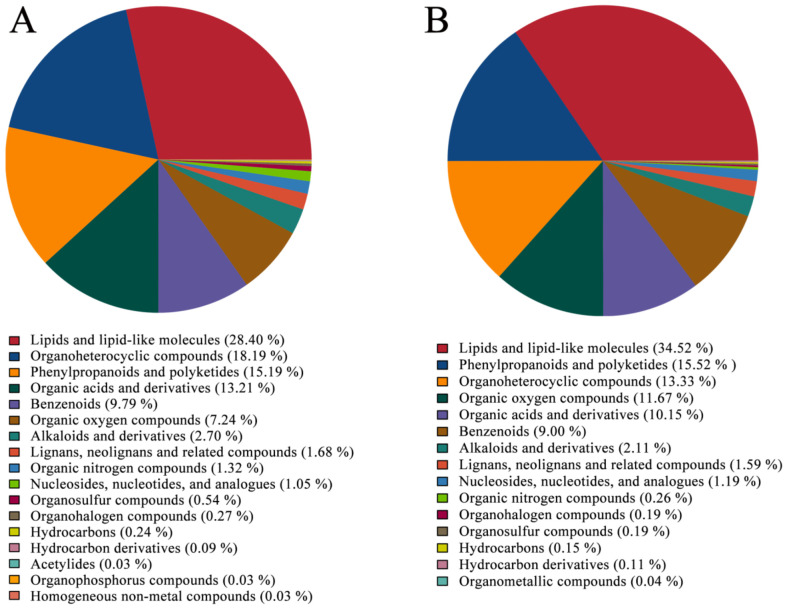
Pie chart of metabolite classification: (**A**) Metabolite classification in positive ion mode. (**B**) Metabolite classification in negative ion mode.

The chemical classification of the identified metabolites in this project was statistically analyzed, and a pie chart was constructed to illustrate the distribution of metabolite classes and the number of compounds in each class. The metabolite classification pie chart revealed that over 60% of the metabolites were classified into the categories of lipids and lipid-like molecules, organoheterocyclic compounds, and phenylpropanoids and polyketides (Figure 3).

### 3.3. Differences in Metabolic Components Among Organs of Understory-Transplanted and Wild D. dentiger

#### 3.3.1. Venn Diagram Analysis of Differential Metabolites

**Figure 4:**
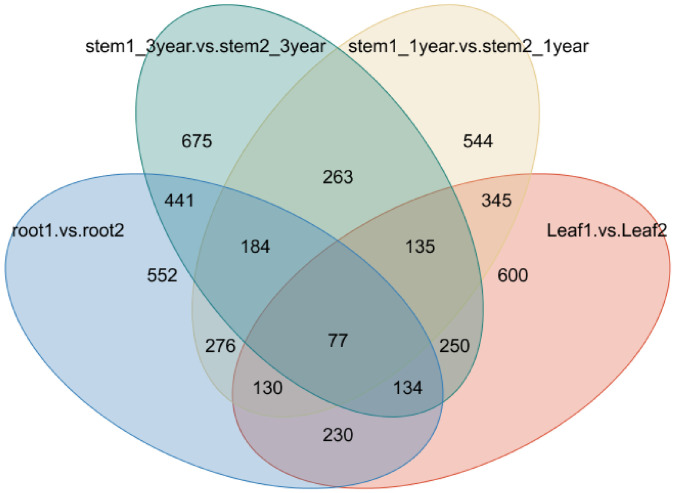
Venn diagram of the number of differential metabolites in metabolic components among organs of understory-transplanted and wild *D. dentiger*.

Analysis of differences in metabolic components among organs of understory-transplanted and wild *D. dentiger* revealed (Table S2 and Figure 2) that there were 1901 differential components in leaves, 1954 in annual stems, 2159 in three-year-old stems, and 2024 in roots (Table 1, Figure 4). Among these, 77 differential metabolites were commonly present across all organs (Table S3). The results indicate that after artificial transplantation, the metabolic components or their contents in various organs of *D. dentiger* are altered.

#### 3.3.2. Differential Metabolite Chord Diagram

**Figure 5:**
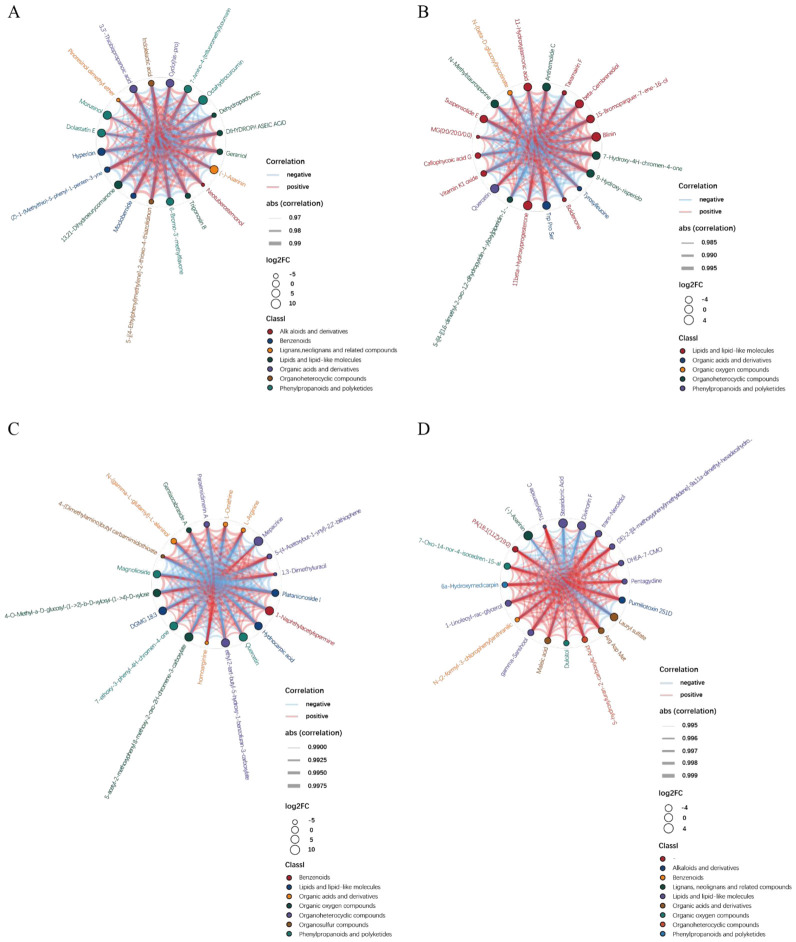
Chord diagram of differential metabolites in metabolic components among organs of understory-transplanted and wild *D. dentiger*: (**A**) Leaf1 vs. Leaf2; (**B**) stem1_1year vs. stem2_1year; (**C**) stem1_3year vs. stem2_3year; (**D**) root1 vs. root2. Dot color represents different classes of metabolites; dot size represents the magnitude of log2 (Fold Change); line thickness between metabolites indicates the strength of correlation; blue lines represent negative correlation, and red lines represent positive correlation.

A chord diagram is a visualization method for displaying correlations among data, in which nodes are arranged radially along the circumference and connected by chords of varying widths. Based on the correlation coefficients of differential metabolites, a chord diagram was constructed to reflect the correlations and degrees of association among differential metabolites in the samples. The top 20 differential metabolites, ranked by ascending *p*-value, were selected for chord diagram presentation. Chord diagrams were generated for differential metabolites in various organs of transplanted and wild *D. dentiger* (Figure 5).

#### 3.3.3. Cluster Diagram of the 77 Differential Metabolites

**Figure 6:**
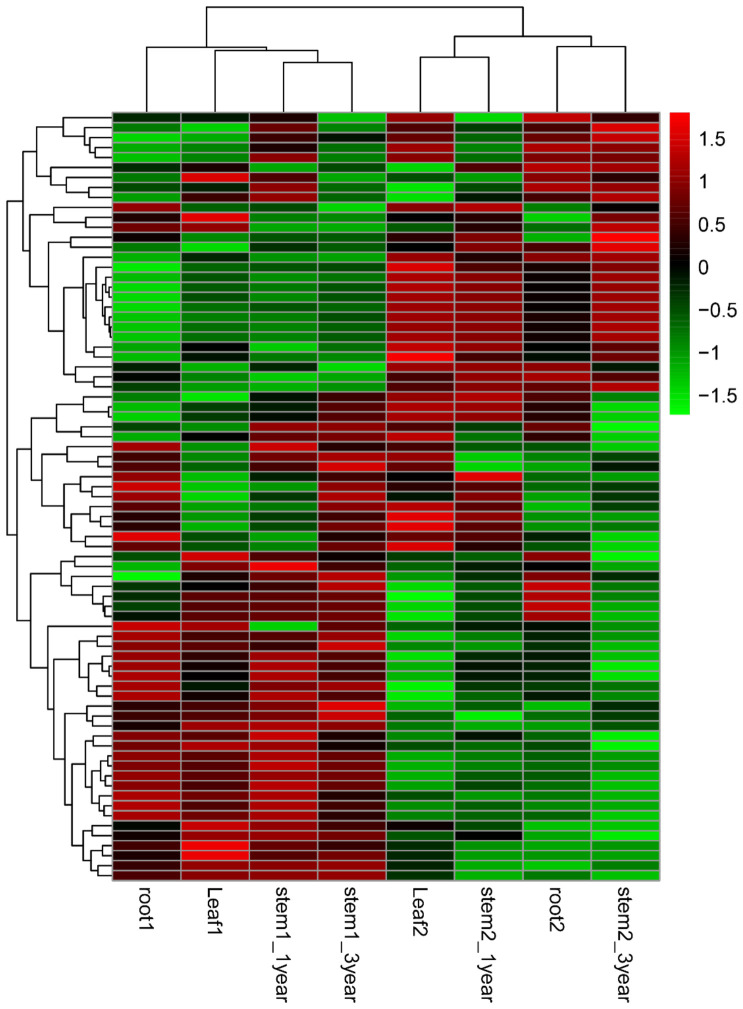
Cluster analysis of differential metabolites in metabolic components among organs of understory-transplanted and wild *D. dentiger*. The vertical axis represents the clustering of samples, and the horizontal axis represents the clustering of metabolites. Shorter cluster branches indicate higher similarity.

Figure 6 presents the cluster diagram of the 77 common differential metabolites screened from various organs of understory-transplanted and wild *D. dentiger*. The relative contents of these components differed among the organs in both habitats. Compared with the top 20 metabolites in the chord diagram, three common metabolites were identified: (Z)-1-(Methylthio)-5-phenyl-1-penten-3-yne (Com_6682_pos), Dihydrophaseic acid (Com_1923_pos), and (-)-Asarinin (Com_3408_pos).

#### 3.3.4. KEGG Classification Analysis

**Figure 7:**
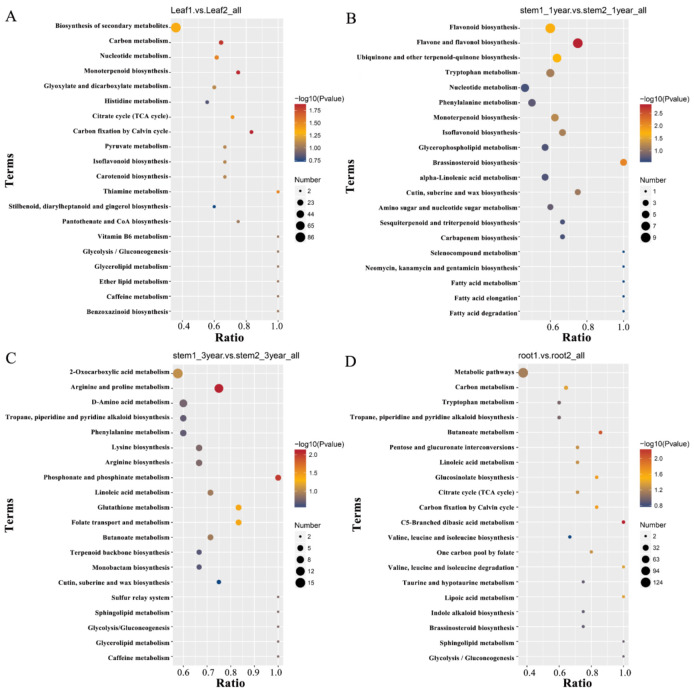
KEGG enrichment bubble plot of differential metabolites among organs of understory-transplanted and wild *D. dentiger*: (**A**) Leaf1 vs. Leaf2; (**B**) stem1_1year vs. stem2_1year; (**C**) stem1_3year vs. stem2_3year; (**D**) root1 vs. root2. The x-axis represents x/y (the number of differential metabolites in the corresponding metabolic pathway divided by the total number of identified metabolites in that pathway). The color of each dot indicates the *p*-value from the hypergeometric test, and the size of the dot represents the number of differential metabolites in the corresponding pathway.

Bubble plots of the KEGG pathways enriched by differential metabolites among various organs of understory-transplanted and wild *D. dentiger* were generated (only the top 20 results are shown). The results revealed that understory transplantation had differential effects on the metabolic component contents in different organs of *D. dentiger*. Specifically, 86 differential metabolites in the leaves of transplanted versus wild *D. dentiger* were enriched in the metabolic pathway “Biosynthesis of secondary metabolites” (Figure 7A); 9 differential metabolites in the annual stems were enriched in “Flavone and flavonol biosynthesis” (Figure 7B); 15 differential metabolites in the three-year-old stems were enriched in “2-Oxocarboxylic acid metabolism” (Figure 7C); and 124 differential metabolites in the roots were enriched in “Metabolic pathways” (Figure 7D).

#### 3.3.5. Analysis of Common Differential Metabolites Across Organs

**Figure 8:**
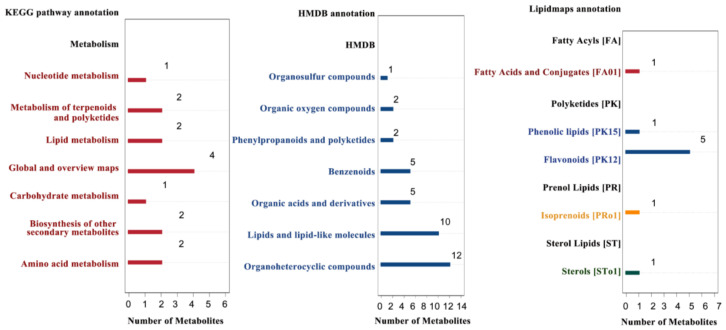
Annotation of the 77 common differential metabolites among organs of understory-transplanted and wild *D. dentiger*.

As shown in Figure 8, the annotation results of the 77 common differential metabolites in the organs of understory-transplanted and wild *D. dentiger* from three databases are summarized. Among them, the most frequently annotated were as follows: 14 compounds were annotated to the KEGG database; 37 compounds were annotated to the HMDB; and 9 compounds were annotated to the LIPID MAPS database (Figure 8). Among these, four compounds were annotated in all three databases: dihydrophaseic acid (Com_1923_pos), stearidonic acid (Com_4297_neg), trifolirhizin (Com_5180_pos), and 3-dehydroteasterone (Com_6863_neg) (Table S4).

#### 3.3.6. Violin Plot of Differential Metabolites

**Figure 9:**
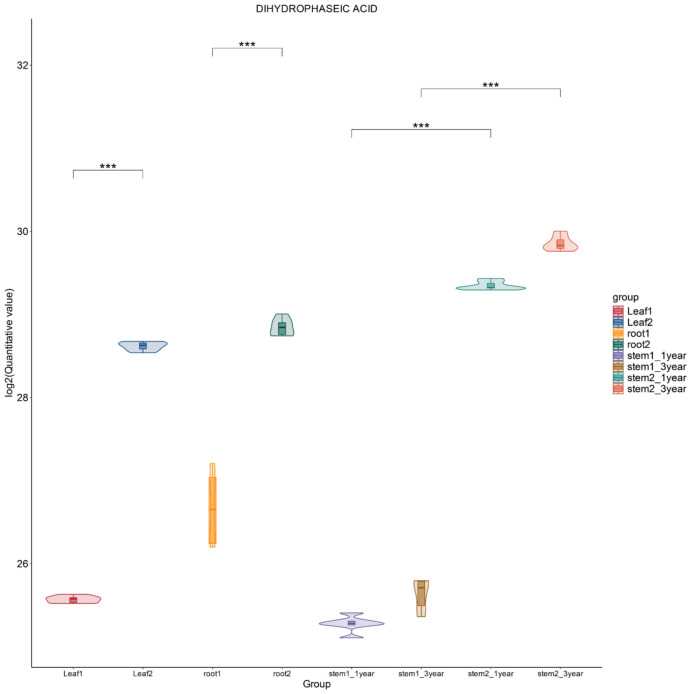
Violin plot of dihydrophaseic acid in each sample of *D. dentiger*. The y-axis represents the quantitative value of the metabolite, and the x-axis represents the different groups. Asterisks indicate the significance of differences between two groups: *** p<0.001.

A total of six metabolites were involved, including the top 20 differential metabolites from the chord diagram, the 77 common metabolites, and the metabolites annotated by all three databases. These six metabolites were: Dihydrophaseic acid (Com_1923_pos), (-)-Asarinin (Com_3408_pos), (Z)-1-(Methylthio)-5-phenyl-1-penten-3-yne (Com_6682_pos), Stearidonic Acid (Com_4297_neg), Trifolirhizin (Com_5180_pos), and 3-Dehydroteasterone (Com_6863_neg). Among them, dihydrophaseic acid (Com_1923_pos) appeared in the chord diagram and was annotated by all three databases. As can be seen from the violin plot of dihydrophaseic acid (Figure 9), its content was relatively high in various organs of wild *D. dentiger* but decreased significantly after understory transplantation. While the contents of Com_3408_pos (Figure S3), Com_6682_pos (Figure S4), and Com_4297_neg (Figure S5) were relatively low in various organs of wild *D. dentiger*, they increased significantly after understory transplantation. However, after understory transplantation, the content changes of Com_5180_pos (Figure S6) and Com_6863_neg (Figure S7) varied across different organs: they decreased in leaves and annual stems but increased in roots and three-year-old stems.

## 4. Discussion

This study compared metabolite profiles across different organs (leaves, annual stems, three-year-old stems, and roots) of wild *D. dentiger* with those of plants subjected to understory transplantation. The results demonstrated that understory transplantation markedly influenced the metabolic composition and metabolite abundance in all organs examined. To further explore organ-specific metabolic responses, KEGG pathway enrichment analyses were performed on differential metabolites between transplanted and wild plants for each organ. The bubble plots (Figure 7) revealed distinct enrichment patterns: in leaves, the “Biosynthesis of secondary metabolites” pathway was enriched; in annual stems, the “Flavone and flavonol biosynthesis” pathway was enriched; in three-year-old stems, “2-Oxocarboxylic acid metabolism” was enriched; and in roots, the “Metabolic pathways” category showed the most pronounced enrichment, with the highest number of differential metabolites (124). This marked enrichment in roots suggests that roots are highly sensitive to changes in the soil environment, likely involving extensive reprogramming of both primary and secondary metabolism. Consistently, previous research has indicated that the chemical composition of plant roots is highly responsive to environmental conditions [20].

Soil heterogeneity (particularly in water availability, nutrient distribution, and physicochemical properties) creates dynamic gradients to which roots must continuously adapt. Importantly, environmental influences on root metabolites are not limited to direct abiotic effects; they are also mediated by the rhizosphere effect, i.e., the bidirectional interactions between roots and soil microbiota [21]. Root exudates shape the structure and metabolism of microbial communities, while microbe-derived signals in turn modulate root development and metabolic profiles [20]. Consequently, the variation in root metabolite composition observed under different growth conditions likely reflects an integrated outcome of abiotic factors and biotic interactions within the rhizosphere. This perspective underscores the necessity for future investigations to dissect the complex relationships among soil properties, soil microbial communities, and root metabolic composition, thereby advancing our understanding of how the rhizosphere environment governs root chemistry and, ultimately, plant performance [22].

Compared with wild plants, the majority of metabolites showed reduced contents (4486 metabolites), while fewer exhibited increases (3552 metabolites; Table 1). Among the 77 common differential metabolites, six (Dihydrophaseic acid, (-)-Asarinin, (Z)-1-(Methylthio)-5-phenyl-1-penten-3-yne, Stearidonic Acid, Trifolirhizin, and 3-Dehydroteasterone) were identified as key metabolites through KEGG enrichment analysis and Metabolite Chord Diagram analysis. As shown in the violin plot (Figure 9 and Figures S3–S7), after understory transplantation, some metabolites consistently decreased (Stearidonic Acid) or increased (Dihydrophaseic acid, (-)-Asarinin, and (Z)-1-(Methylthio)-5-phenyl-1-penten-3-yne) across all organs, while others (Trifolirhizin and 3-Dehydroteasterone) exhibited organ-dependent changes, decreasing in leaves and annual stems but increasing in roots and three-year-old stems. Collectively, these findings demonstrate that the cultivation environment significantly affects the metabolite composition and abundance in medicinal plants, consistent with observations reported for *Chaenomeles speciosa* [23].

Dihydrophaseic acid (DPA) is the stable oxidative metabolite of abscisic acid (ABA) formed via the 8′-hydroxylation pathway [24]. It is generally considered an inactive ABA catabolite. In plant stress responses, DPA levels increase under drought stress [25]. This compound is widely distributed across plant species and organs, with tissue-specific differences in ABA metabolism rates determining its accumulation [26]. To date, reports on the biological functions of (Z)-1-(Methylthio)-5-phenyl-1-penten-3-yne are extremely limited, and its specific physiological activities as well as its role in plant metabolism remain to be further explored.

(-)-Asarinin is a tetrahydrofurofurano lignan with broad pharmacological activities, including anti-allergic, anti-inflammatory, anticancer, neuroprotective, antioxidant, antiviral, and analgesic effects [27]. Network pharmacology studies have revealed that (-)-Asarinin can induce apoptosis, thereby alleviating gastric precancerous lesions, and also exhibits anti-migraine activity [28,29]. Stearidonic acid (SDA, 18:4n-3) is a Δ6-unsaturated C18 omega-3 polyunsaturated fatty acid [30]. Studies have shown that SDA is predominantly present in a limited number of plant species, typically represented by the Boraginaceae and Primulaceae families, reflecting the general lack of Δ6-desaturase activity in higher plants [21]. Lipids containing SDA hold significant application potential in areas such as fortified foods, dietary supplements, medical foods, and pharmaceuticals [20]. Consequently, recent efforts have also attempted to achieve efficient SDA accumulation in plants such as *Camelina sativa* through transgenic approaches, thereby offering the possibility of developing novel plant-derived supplements [31].

Therefore, it is worth considering the extraction of (-)-Asarinin and Stearidonic acid from *D. dentiger* plants transplanted to the understory, as their contents are higher than those in wild plants. Understory transplantation significantly altered the metabolic profiles of various organs of *D. dentiger*, with distinct response patterns among different organs. Metabolites such as dihydrophaseic acid may serve as indicators for monitoring recovery from transplantation stress, providing a metabolomics basis for the artificial cultivation and quality control of *D. dentiger*. However, this study did not perform absolute quantification of metabolites, and the results are representative of only a single time point. Future research should therefore consider comparisons across multiple environmental sites or different stand densities.

## 5. Conclusions

This study conducted a comparative untargeted metabolomic analysis of different organs (roots, stems, and leaves) of *Dendropanax dentiger* between understory-transplanted and wild conditions. The results revealed significant differences in metabolite composition, with three bioactive compounds ((-)-asarinin, (Z)-1-(methylthio)-5-phenyl-1-penten-3-yne, and stearidonic acid) being higher in transplanted plants than in wild plants. These findings demonstrate that understory cultivation can effectively enhance the accumulation of certain medicinal components in *D. dentiger*.

## Figures and Tables

**Table 1:** Screening results of differential metabolites in metabolic components among organs of understory-transplanted and wild *D. dentiger*.

Compared Samples	Num. of Total Sig.	Num. of Sig.Up	Num. of Sig.down
Leaf1 vs. Leaf2	1901	893	1008
stem1_1year vs. stem2_1year	1954	782	1172
stem1_3year vs. stem2_3year	2159	1131	1028
root1 vs. root2	2024	746	1278

## Data Availability

The original contributions presented in this study are included in the article. Further inquiries can be directed to the corresponding author.

## Supplementary Materials

The following supporting information can be downloaded at: https://www.mdpi.com/article/10.3390/metabo16060354/s1, Figure S1: The representative LC–MS chromatograms for each sample group in positive ion mode; Figure S2: The representative LC–MS chromatograms for each sample group in negative ion mode; Figure S3: Violin plot of Asarinin in each sample of *D. dentiger*; Figure S4: Violin plot of (Z)-1-(Methylthio)-5-phenyl-1-penten-3-yne in each sample of *D. dentiger*; Figure S5: Violin plot of Stearidonic Acid in each sample of *D. dentiger*; Figure S6: Violin plot of Trifolirhizin in each sample of *D. dentiger*; Figure S7: Violin plot of 3-Dehydroteasterone in each sample of *D. dentiger*; Table S1: Functional and classification annotation of 6185 compounds (3423 in positive ion mode and 2762 in negative ion mode) using HMDB, KEGG and LIPID MAPS metabolite databases; Table S2: Analysis of differences in metabolic components among organs of understory-transplanted and wild *D. dentiger*; Table S3: The functional and classification annotation of 77 differential metabolites; Table S4: The annotation of 77 differential metabolites using HMDB, KEGG and LIPID MAPS metabolite databases.
