# Supplementary figures and images for: Comparative Metabolomic Analysis of Different Organs of Understory-Transplanted and Wild Dendropanax dentiger
Source: Metabolites. 2026 May 25;16(6):354. doi: 10.3390/metabo16060354 (PMC13304204; doi:10.3390/metabo16060354)

(-)-Asarinin

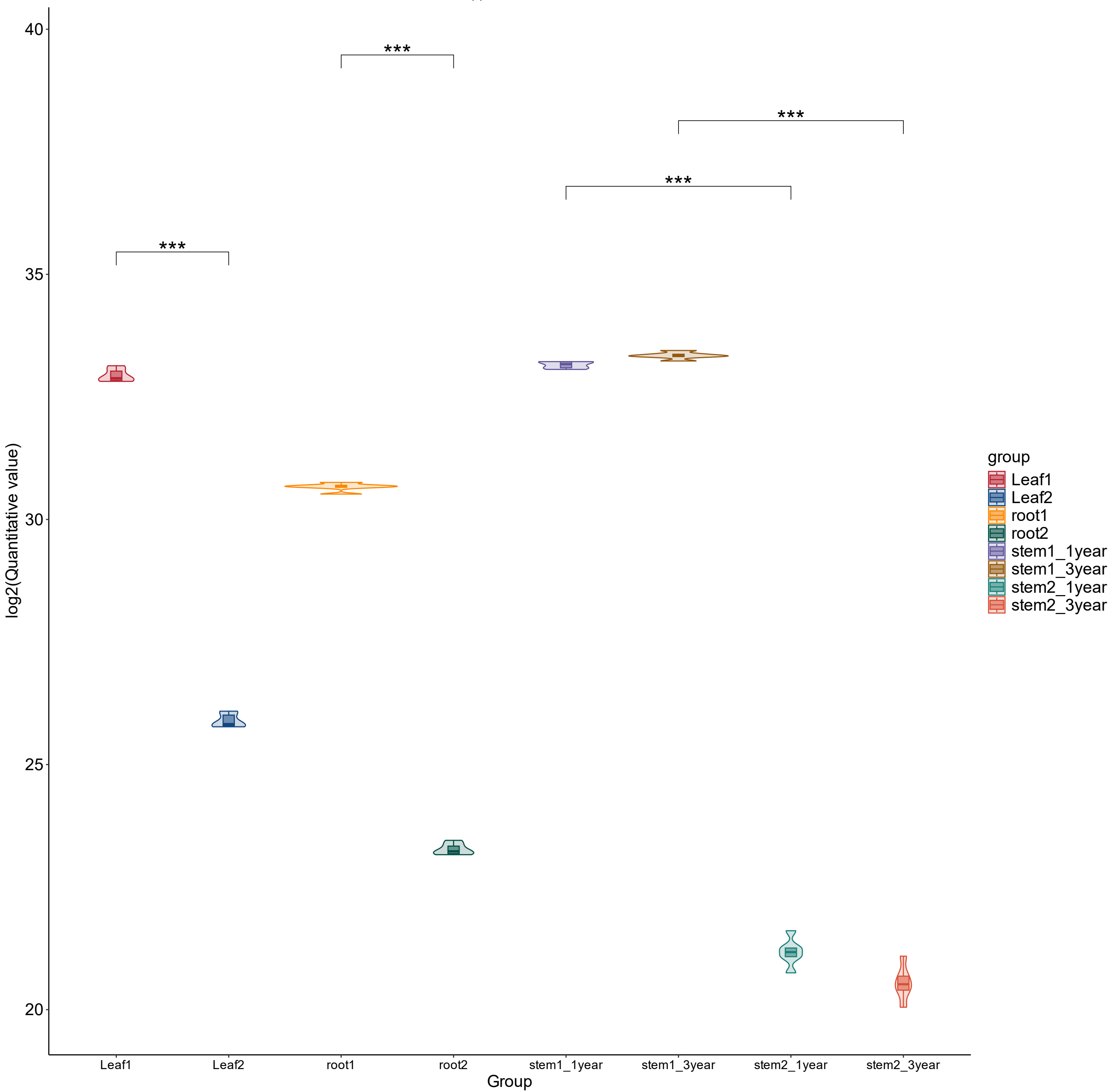

Supplement: Supplementary file 1 [file metabolites-16-00354-s001.zip › Figure S3.pdf]

(Z)-1-(Methylthio)-5-phenyl-1-penten-3-yne

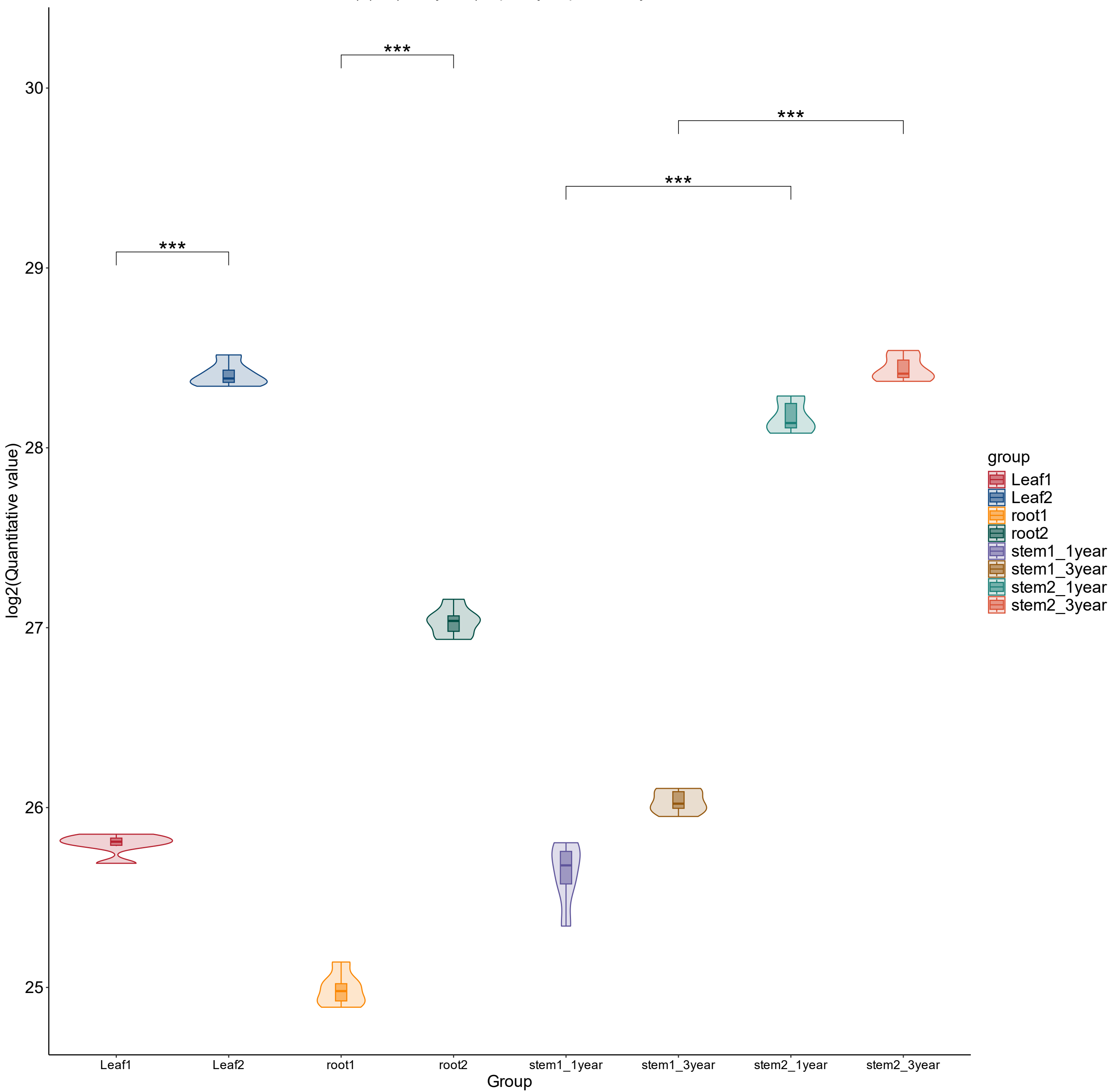

Supplement: Supplementary file 1 [file metabolites-16-00354-s001.zip › Figure S4.pdf]

# Stearidonic Acid

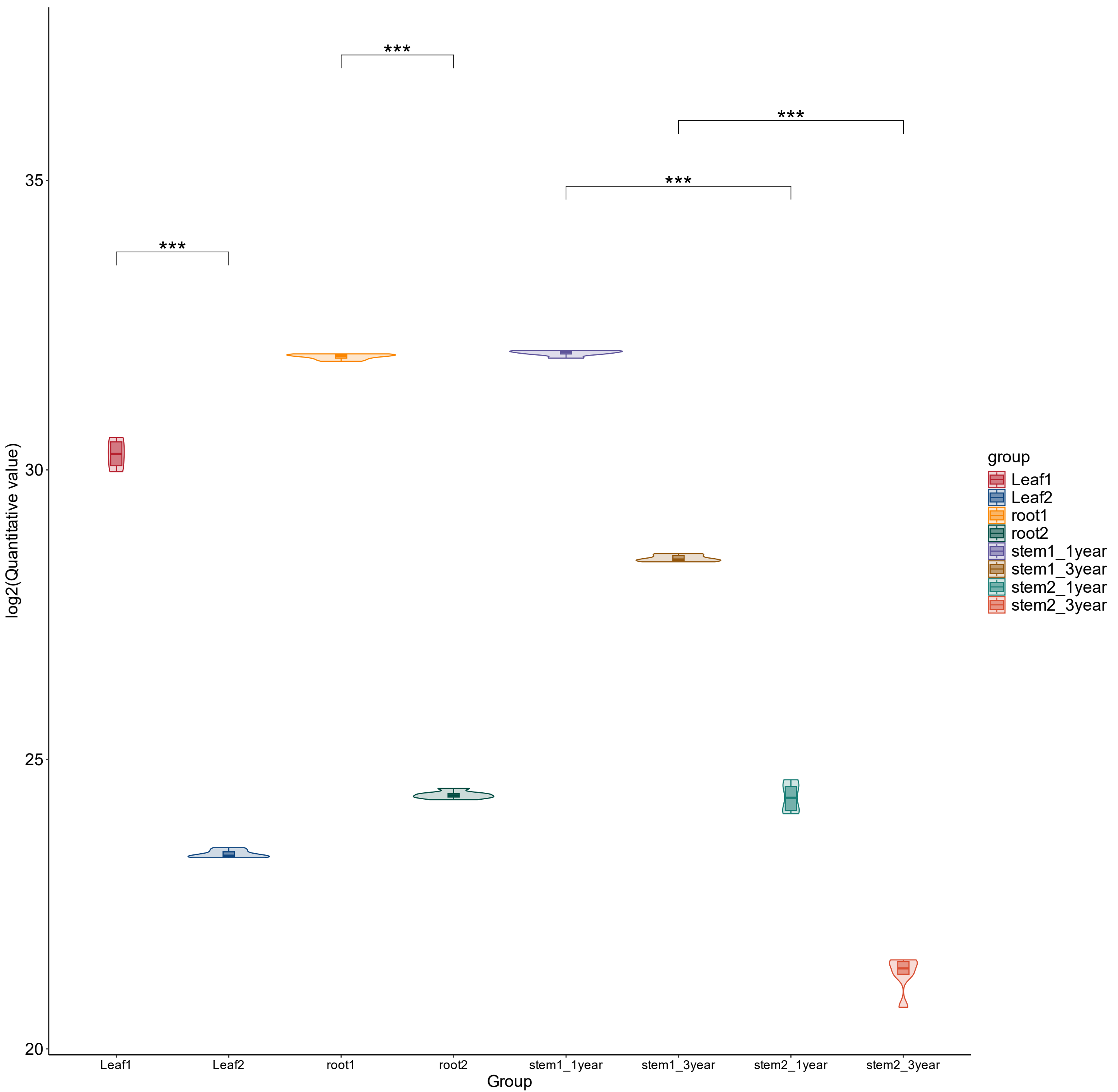

Supplement: Supplementary file 1 [file metabolites-16-00354-s001.zip › Figure S5.pdf]

# Trifolirhizin

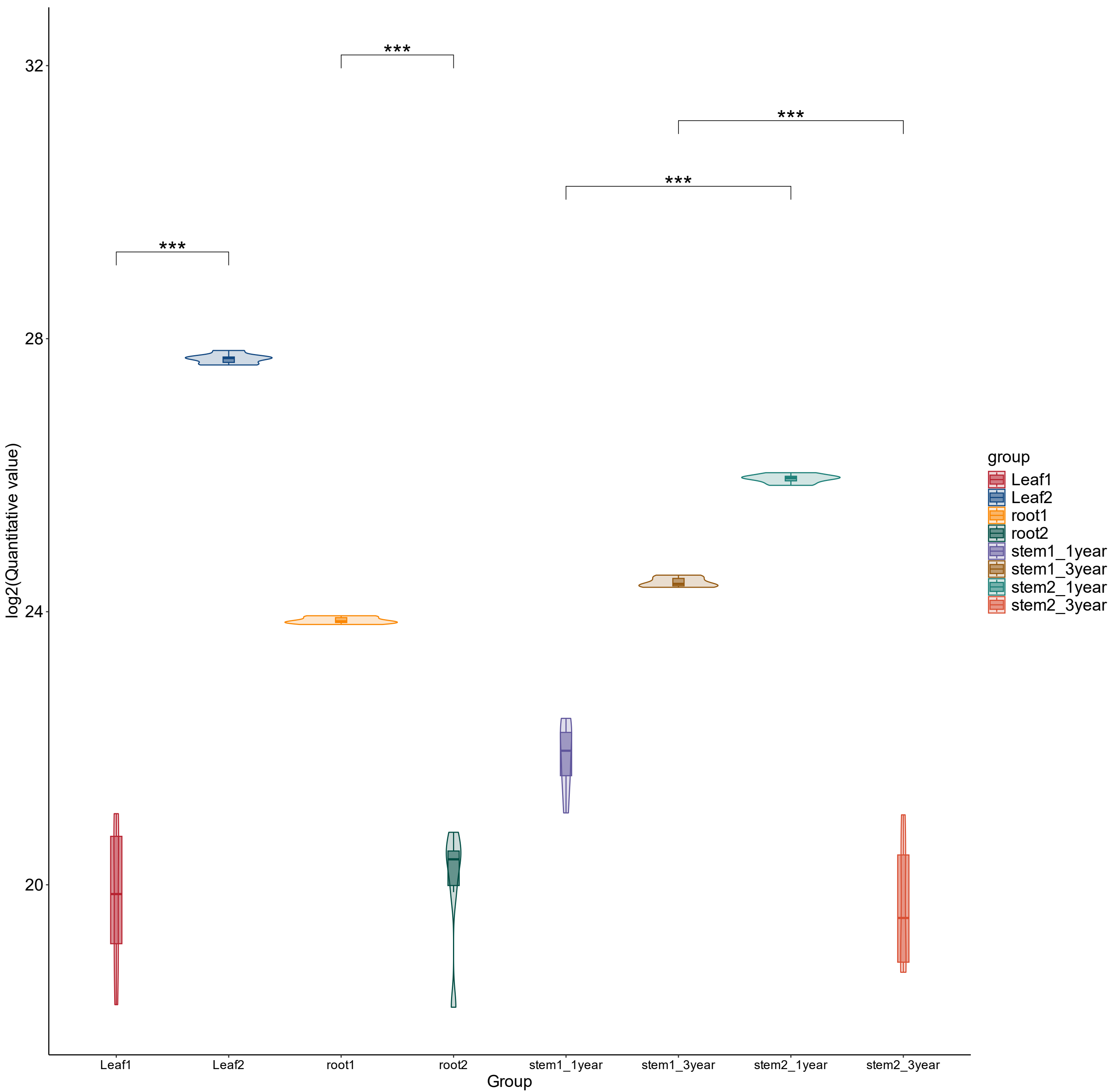

Supplement: Supplementary file 1 [file metabolites-16-00354-s001.zip › Figure S6.pdf]

# 3-Dehydroteasterone

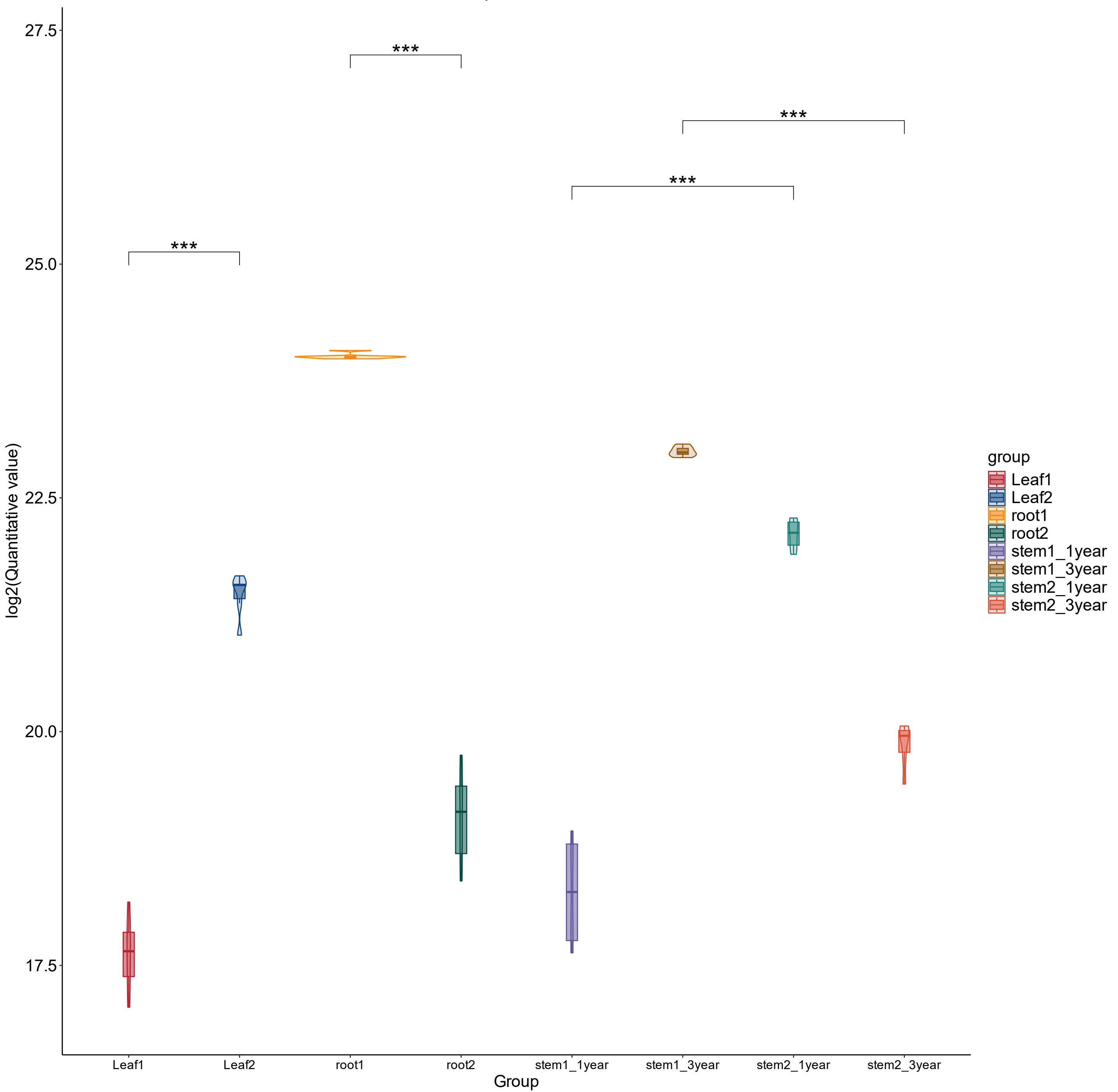

Supplement: Supplementary file 1 [file metabolites-16-00354-s001.zip › Figure S7.pdf]
